# Performance Study of a Leaf-Vein-like Structured Vapor Chamber

**DOI:** 10.3390/ma16124482

**Published:** 2023-06-20

**Authors:** Zhihao Zhou, Xu Wang, Yongmin Zhou

**Affiliations:** College of Materials Science and Engineering, Nanjing Tech University, South Puzhu Road No. 30, Nanjing 211816, China; 202061203279@njtech.edu.cn (Z.Z.); 202061203241@njtech.edu.cn (X.W.)

**Keywords:** vapor chamber, leaf vein, cotton thread, natural convection, heat dissipation

## Abstract

As optoelectronic products continue to advance rapidly, the need for effective heat dissipation has become increasingly crucial due to the emphasis on miniaturization and high integration. The vapor chamber is widely used for cooling electronic systems as a passive liquid–gas two-phase high-efficiency heat exchange device. In this paper, we designed and manufactured a new kind of vapor chamber using cotton yarn as the wick material, combined with a fractal pattern layout of leaf veins. A comprehensive investigation was conducted to analyze the performance of the vapor chamber under natural convection circumstances. SEM showed that many tiny pores and capillaries were formed between the cotton yarn fibers, which are very suitable as the wick material of the vapor chamber. Additionally, experimental findings demonstrated the favorable flow and heat transfer characteristics of the cotton yarn wick within the vapor chamber, which makes the vapor chamber have significant heat dissipation capability, compared to the other two vapor chambers; this vapor chamber has a thermal resistance of only 0.43 °C/W at a thermal load of 8.7 W. In addition, the vapor chamber showed good antigravity capability, and its performance did not show significant changes between horizontal and vertical positions; the maximum difference in thermal resistance at four tilt angles is only 0.06 °C/W. This paper also studied the influence of vacuum degree and filling amount on the performance of the vapor chamber. These findings indicate that the proposed vapor chamber provides a promising thermal management solution for some mobile electronic devices and provides a new idea for selecting wick materials for vapor chambers.

## 1. Introduction

As information technology, electronics, and related industries continue to progress rapidly, a wide range of electronic products have emerged, such as smartphones, tablet PCs, etc., which are developing in the direction of miniaturization, integration, and lightweight. The increase in heat generation not only has a significant impact on the life of electronic devices and the stability of use, but too high a surface temperature will also affect the user experience. A vapor chamber, commonly referred to as a flat heat pipe [1,2,3], is a specialized heat sink extensively utilized in electronic products. The vapor chamber has attracted significant attention from researchers due to its unique characteristics compared to heat pipes. These include a large condensation area, uniform surface temperature distribution, and wide range of applications, among others [4,5,6]. The operational mechanism of a vapor chamber closely resembles that of a heat pipe. At the evaporation end of the vapor chamber, the working fluid within the wick absorbs heat, causing it to heat up and evaporate. The vapor condenses when it comes into contact with the colder wall due to the rapid movement of vapor pressure steam around. Consequently, the working fluid releases its latent heat of vaporization, and through capillary action, the condensed fluid returns to the heat source.

Vapor chambers have been widely explored in recent years due to their superior heat homogenization performance. In order to improve the performance of vapor chambers, many researchers are exploring various new wick materials and structures. Tang et al. [7] manufactured a vapor chamber that utilizes sintered copper powder as the wick material and employs a porous cylindrical structure as a support pillar. Their study indicates that this vapor chamber exhibits a minimum thermal resistance of 0.08 K/W and a maximum heat transfer limit of 300 W/cm^2^. Ji et al. [8] utilized porous copper foam as a wick material in the vapor chamber and conducted experiments to determine that the minimum thermal resistance of this vapor chamber can reach 0.09 K/W. Furthermore, it was found to operate effectively under a heat flux density of 216 W/cm^2^. Yu et al. [9] utilized an electrodeposition method to fabricate porous copper material, which was subsequently applied in a vapor chamber. Experimental results demonstrated that the vapor chamber with this electrodeposited copper core exhibited excellent heat dissipation capabilities. Peng et al. [10] introduced a unique leaf vein microchannel slot wick system in a vapor chamber. The design of this vapor chamber, created through chemical etching, facilitated enhanced temperature uniformity and minimized thermal resistance. These benefits are attributed to the enhanced fluid flow and increased condensation area resulting from this structural configuration. Wong et al. [11,12] developed a new type of vapor chamber by engraving microgrooves on the condenser-side shell plate and using a sintered core as the liquid wick on the evaporator-side shell plate. Assessment of this novel vapor chamber demonstrated a lower thermal resistance compared to conventional counterparts. Wang et al. [13] investigated and studied the heat dissipation performance of groove-type and foam copper-type vapor chambers. The results showed that the thermal resistance of the foam copper-type vapor chamber is lower than that of the groove-type vapor chamber because the foam copper can provide greater capillary force. Yang et al. [14] integrated a wetting-patterned substrate underneath a nanoscale-structured mesh core, and experimentally evaluated the thermal resistance of the vapor chamber. The measurement findings revealed that the utilization of a wetting-patterned substrate beneath the nanoscale-structured mesh core led to a notable reduction in horizontal thermal resistance. Furthermore, this structure also enables a more uniform surface temperature on the condenser side of the vapor chamber. Liu et al. [15] utilized a metal wire mesh as the wick in the vapor chamber and applied a super hydrophilic surface structure to the mesh. This structure significantly improved the thermal performance of the vapor chamber. Yu et al. [16] fabricated a vapor chamber with a spiral woven mesh using a brazing process, and the maximum heat transfer capacity of the vapor chamber was experimentally determined to be 6 W.

Many researchers are not satisfied with the heat dissipation performance of a single-structured wick vapor chamber and have further employed various methods to enhance its heat dissipation performance. Deng et al. [17] fabricated a composite wick structure vapor chamber and engraved numerous small grooves on the evaporator-side shell plate, and analysis showed that it had good temperature uniformity and thermal performance. Shaeri et al. [18] fabricated an innovative vapor chamber to assess the viability of integrating hydrophobic and hydrophilic properties in the evaporator for enhanced thermal performance. The findings demonstrated that the proposed vapor chamber exhibited a lower thermal resistance compared to conventional designs. Ju et al. [19] developed a novel vapor chamber with two types of core structures designed internally. The single-layer sintered copper powder core effectively reduces the thermal resistance of the vapor chamber. They also compared the performance of three different core structures, including vertical columnar artery, convergent lateral artery, and double-hole system. Patankar et al. [20] devised a methodology for designing vapor chambers specifically tailored to meet the distinct transport constraints and thermal management conditions to be met in mobile electronics. The vapor chamber was intended to improve condenser-side temperature uniformity in an ultrathin shape mainly by controlling the condenser-side core to improve lateral heat transfer. Zhao et al. [21] developed a composite wick vapor chamber by sintering copper mesh and powder. Through experimental analysis, it was determined that the vapor chamber achieved a maximum heat dissipation power of 23.29 W, while maintaining a minimum thermal resistance of 0.33 °C/W. Sudhakar et al. [22] designed a dual-layer wick structure in the vapor chamber. Experimental results showed that, compared to the single-layer wick structure vapor chamber, it could withstand higher heat flux densities. George et al. [23] designed a vapor chamber without a wick structure in which the internal work fluid flows using the surface tension of the wettability pattern inside the shell plate. The thermal resistance of this vapor chamber was found to be 0.18 K/W at 10 W by experimental test results.

Through billions of years of evolution, nature has provided us with opportunities to learn from its various behaviors and physiological structures across different domains. Inspired by leaf veins, Liu et al. [24] designed and engraved a leaf-vein-like groove network on the condenser side of the vapor chamber, while using a sintered copper core as the wick on the evaporator side. Experimental results demonstrated that this vapor chamber exhibited excellent performance. Peng et al. [25,26] fabricated a new vapor chamber wick structure based on a fractal network of plant veins, comprising a grooved design. The findings demonstrated that the system was characterized by sizeable capillary pressure and high penetration; therefore, the vapor chamber has good performance. However, the design and fabrication of this wick were complex, and the fabrication of fine grooves required costly chemical etching.

Inspired by the water transport capacity of plant leaves, a new biomimetic vapor chamber was designed and manufactured in this study. The vapor chamber used an aluminum alloy as its shell and cotton yarn as the wick material. The fractal structure of leaf veins was imitated to design the distribution of the wick material, and the heat dissipation performance of the vapor chamber under natural convection conditions was studied through experimental testing.

## 2. Design and Fabrication

### 2.1. Production of Vapor Chamber

The structure of the internal suction wick of the vapor chamber and the fabrication method of the vapor chamber are described in this section. The upper and lower shells of the vapor chamber formulated in this study were made of 6061 aluminum alloy with 160 × 80 × 1 mm as the dimensions. Anhydrous ethanol was used as the working fluid. To create the cavity structure of the vapor chamber, an aluminum wire with a diameter of 2 mm was bent into a 160 × 80 mm rectangle as the intermediate frame of the vapor chamber, and then the upper and lower shell plates were bonded together with the middle frame using metal adhesive to form the cavity structure. The surrounding area of the bonding was further sealed with organic silicon sealant. The method used to make the vapor chamber in this study was simple, and the vapor chamber can be disassembled and reused for other studies. This significantly reduces the cost of fabrication and saves resources. The roles of the primary materials are shown in Table 1. The main production process includes cleaning the upper and lower shell plates, arranging and fixing the wick, assembling the upper and lower shell plates, adding liquid-filled tubes, leak detection, liquid-filling, vacuuming, and sealing the liquid-filled tubes.

### 2.2. Structural Design of the Wick

**Figure 1:**
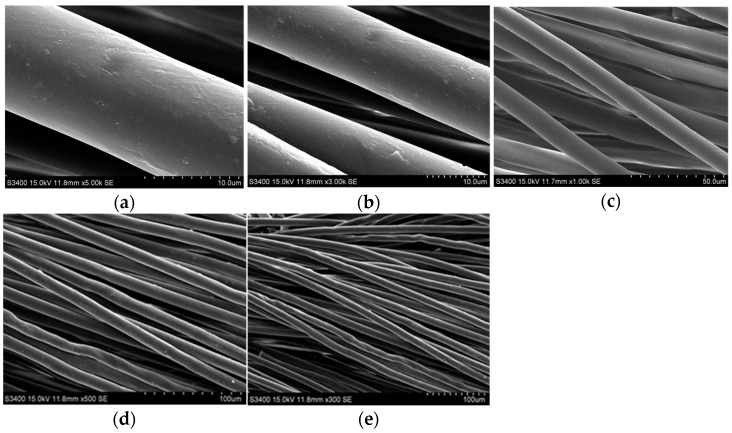
SEM image of the wick: (**a**) 5000 times; (**b**) 3000 times; (**c**) 1000 times; (**d**) 500 times; (**e**) 300 times.

Findings on the transpiration of plants and previous studies on plant leaf veins [27,28] indicate that the fractal structure of leaf veins has a high axial conductivity. This characteristic of the fractal structure allows water transport to the leaf’s distal end with low water potential loss. This naturally optimized structure was mimicked to design the wick inside the vapor chamber. The wick structure developed in this study was made of cotton woven from cotton. Cotton is a natural fiber with good moisture absorption. Moreover, the woven cotton thread has good flexibility [29] and can be designed into various desired shapes, which can be utilized as the wick of the vapor chamber. Figure 1 shows the scanning electron microscope image of cotton thread; the surface of the cotton thread is formed by layers of interwoven fibers, which create many tiny pores and capillaries between the fibers. These features are crucial for efficiently transporting the working fluid in the vapor chamber. When the vapor chamber is activated, the strong capillary forces drive the working fluid to flow along the tiny pores formed between the cotton thread fibers toward the heat source, facilitating the internal phase-change heat transfer process.

**Figure 2:**
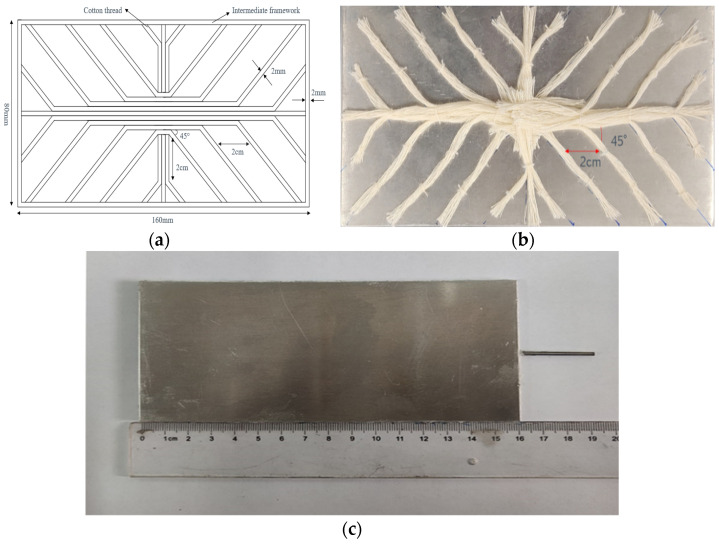
(**a**) Schematic diagram of wick; (**b**) physical diagram of wick; (**c**) finished vapor chamber.

A schematic diagram showing the design process for obtaining the wick is shown in Figure 2a. The fractal points were 2 cm apart, and the fractal angle was 45°. The structure of the wick made by this design is shown in Figure 2b. According to the above method, 24 strands of cotton thread are required to arrange the wick inside the vapor chamber. As the vapor chamber has different lengths and widths, an 8.5 cm cotton yarn was used in the length direction, and a 4.5 cm cotton yarn was used in the width direction, resulting in a 10 × 10 mm intersection area in the center of the shell plate. The heat source is located below the intersection area. Each branch has a strand of cotton thread. The diameter of the cotton thread was 2 mm (made of 19 fine cotton yarn).

According to the above design, the fixation method used a marker pen to draw the fractal network on the shell plate. Then, using adhesive, fine yarn with a diameter of 0.1 mm was fixed at each branching point of the vapor chamber shell plate. The distance between each fixed point was 2 cm. Finally, the cotton yarn was tied to the shell plate according to the fractal structure using fine thread. This process was conducted to ensure the integrity of the internal capillary tube of the wick. Fixing the cotton yarn using dispensing would block the capillary hole and obstruct the working fluid return. Because no suitable method has been found to restore the cotton thread on the shell plate, only this manual method can be used to fix it.

After securing the wick, to prevent deformation under vacuum conditions, support columns were incorporated into the design of the vapor chamber, providing essential structural support. The support column is made of nylon and is a cylinder with a diameter of 5 mm and a length of 2 mm. The upper and lower shell plates, the middle frame, and the liquid-filled tube are assembled using the method described in Section 2.1. The completed finished product is shown in Figure 2c. The working fluid is injected through the filler tube, and the cavity is evacuated using a vacuum pump. Finally, the end of the filler tube is flattened to form a temporary cold weld seal.

## 3. Experimental Setup and Methods

### 3.1. Experimental Setup

**Figure 3:**
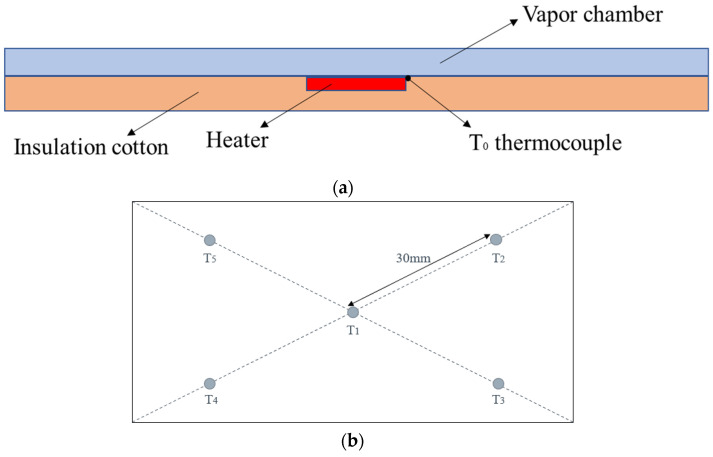
(**a**) Testing bench of vapor chamber; (**b**) condensing end surface thermocouple distribution map.

Figure 3a shows the schematic of the experimental setup used to evaluate the heat transfer performance of the vapor chamber samples designed in this study under natural convection conditions. The heating module was made of a ceramic heating block of 10 × 10 × 2 mm. This module had the advantages of fast heating speed and uniform heat distribution, and its heating power could be adjusted by adjusting the voltage of the connected DC power supply. To minimize heat loss, the heating block was positioned at the central bottom of the vapor chamber and surrounded by insulation cotton. Additionally, a thin layer of thermal grease was applied to the contact surface between the heating block and the vapor chamber, effectively reducing contact thermal resistance. The thermocouples were type K (−20–200 °C) and were calibrated to an uncertainty of ±0.1 °C; this range covered the anticipated temperature fluctuations during the operation of the vapor chamber. The chosen precision level was sufficient to meet experimental needs. Thermocouple T_0_ was placed at the edge of the heat source in close proximity to the vapor chamber shell to approximate the heat source’s surface temperature and the vapor chamber’s evaporator temperature, as shown in Figure 3a. Figure 3b shows the distribution of thermocouples on the condenser surface. The room temperature was approximately around 20 °C during the experiments. However, it is important to note that we did not deliberately control or regulate the room temperature; therefore, the room temperature may fluctuate slightly during the experiment.

### 3.2. Data Reduction and Uncertainty Analysis

The input power of the heat source is expressed as follows:

(1) Q=UI

The input power of the heat source was determined by multiplying the readings displayed on the DC power meter, denoted as *U* and *I*, which were connected to the heating block.

The maximum temperature difference $\Delta T_{max}$ at the surface of the condensing end of the vapor chamber is expressed as $\Delta T_{max}=\max\left\{T_{1},T_{2},T_{3},T_{4},T_{5}\right\}-\min\left\{T_{1},T_{2},T_{3},T_{4},T_{5}\right\}$, where $T_{1}$, $T_{2}$, $T_{3}$, $T_{4}$, $T_{5}$ represent the temperatures measured by the thermocouples on the surface of the condensing end.

The thermal resistance *R* of the vapor chamber is expressed as follows:

(2) $$R=\frac{T_{0}-T_{average}}{Q}$$

where $T_{0}$ denotes the temperature measured by the thermocouple and $T_{average}$ represents the average temperature of the surface at the condensing end of the vapor chamber; $T_{average}$ can be expressed as $T_{average}=\frac{T_{1}+T_{2}+T_{3}+T_{4}+T_{5}}{5}$.

The primary sources of uncertainty in the test system can be attributed to the adjustable DC power supply and the K-type thermocouple. The adjustable DC power supply has an accuracy of 0.5% for current measurements and 0.1% for voltage measurements. The calibration measurement accuracy of the type K thermocouple was 0.1 °C, and the error of the data logger was 0.5%. The measurement uncertainties were analyzed using the standard error analysis method. The maximum relative uncertainties for the input power and thermal resistance were 0.1% and 6.5%, respectively.

## 4. Results and Discussion

### 4.1. Performance Comparison of Different Core Structures

This section compares the heat transfer performance among three different vapor chambers. VC1 has no wick inside, VC2 is the vapor chamber designed in this study, and VC3 has a wick made of cotton threads with the same mass as VC2, randomly distributed in the vapor chamber’s cavity. The dimensions of the three vapor chambers are 160 × 80 × 4 mm, with a filling amount of 1 g and an absolute pressure of 0.028 MPa in the empty hole.

**Figure 4:**
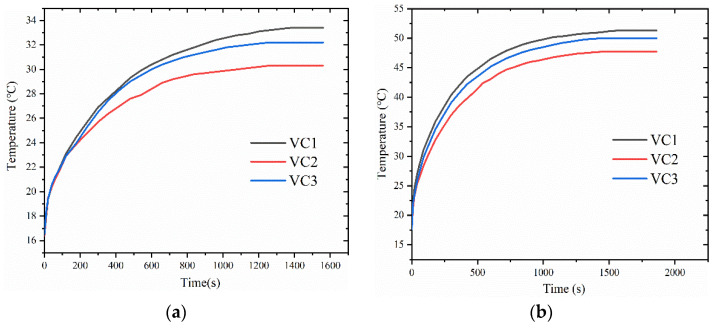

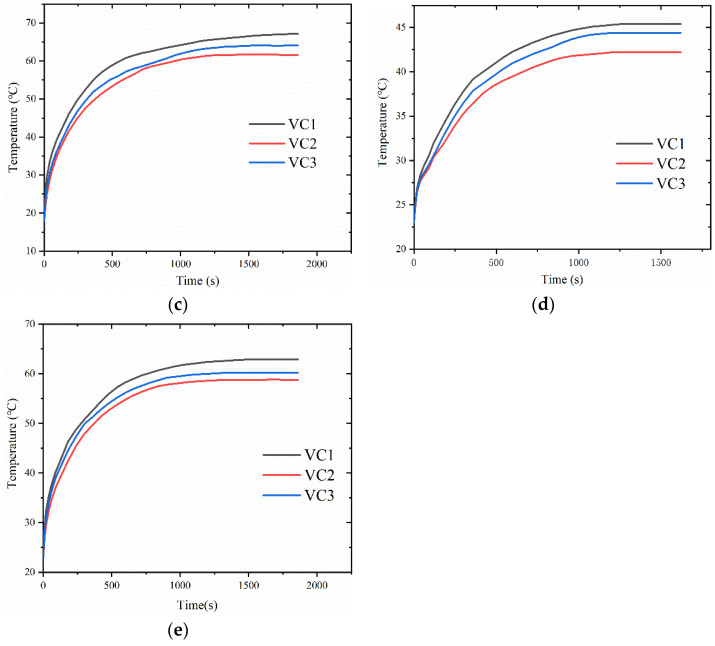
Heat source temperature of different structure of the vapor chamber under different heat generating power: (**a**) 2.6 W; (**b**) 5.8 W; (**c**) 8.7 W; (**d**) 4.1 W; (**e**) 6.9 W.

The three vapor chambers were tested at heating powers of 2.6 W, 4.1 W, 5.8 W, 6.9 W, and 8.7 W, respectively. Figure 4 shows the connection between the surface temperature of the heat source and time for the three types of vapor chambers. From the figure, it can be found that the temperature of the heat source corresponding to all three vapor chambers increases with the increase of heat load. The time taken by VC1 to reach stability is the longest, and the stabilization time is 1380 s, 1440 s, 1500 s, 1560 s, and 1740 s, respectively, for the three heating powers. Furthermore, the surface temperature of the heat source is the highest after stabilization. The stabilization time of VC2 is the shortest, and it is 1260 s, 1320 s, 1380 s, 1380 s, and 1440 s, respectively, for the five heating powers. VC2 has the lowest surface temperature of the heat source after stabilization and VC3 is in between the two. This may be because VC1 has no wick, and the working fluid in the cavity of the vapor chamber can only return to the heat source through gravity and natural flow after being heated and evaporated, resulting in the drying out of the heat source area and seriously damaging the performance of the vapor chamber. Although VC3 has a wick, its random distribution obstructs the flow of working fluid and steam, affecting its performance. The wick of VC2 is made of a leaf vein fractal structure, which is conducive to the flow of working fluid inside and enables the condensate, after being heated and evaporated, to quickly return to the heat source through the wick at the condensation end of the vapor chamber.

**Figure 5:**
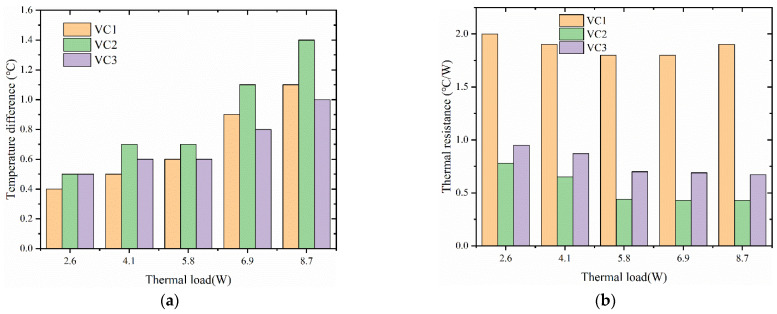
Surface temperature difference and thermal resistance of different structures of vapor chamber under different heat generating power: (**a**) surface temperature difference of vapor chamber; (**b**) thermal resistance of vapor chamber.

Figure 5 shows the thermal resistance and condenser surface temperature difference of the three vapor chambers after reaching a steady state. From Figure 5a, it can be seen that the maximum surface temperature difference at the condensing end of the three vapor chambers increases with the growth of the thermal load. The surface temperature difference of VC2 is more significant than the other two under all five heating powers. This may be because the wick of VC2 converges at the center position of the vapor chamber, connecting the upper and lower shell plates. At the same time, the heat source is heated at the bottom, forming another heat transfer path: the heat is transferred to the evaporative end shell plate through the wick and the working fluid inside the wick, which results in a greater surface temperature difference than the other two structures of the vapor chamber. As seen in Figure 5b, the thermal resistance of VC1 is the largest, followed by VC3, and VC2 has the most minor thermal resistance. This is because in VC1, the condensate returns too slowly after the working fluid evaporates, causing drying out of the heat source area and failure of the vapor chamber, resulting in an increase in thermal resistance. This further demonstrates that the vapor chamber with leaf vein fractal structure designed in this study has excellent fluid flow and heat convection performance.

### 4.2. Empty Cavity Pressure’s Effect on Vapor Chamber Performance

**Figure 6:**
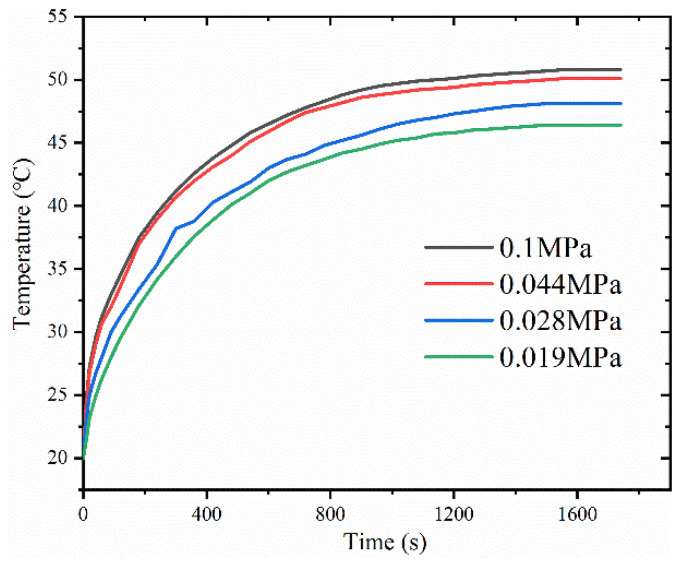
Variation of surface temperature of heat source with time under different pressure of vapor chamber.

Figure 6 shows the variation of the temperature of the heat source surface with time at different pressures inside the vapor chamber, which was tested under a heat load of 5.8 W with a filling amount of 1 g. The results indicate that the vapor chamber with lower pressure has a shorter startup time, as indicated by the smaller slope at the beginning and a lower temperature after reaching a steady state. Specifically, the heat source temperature at pressures of 0.1 MPa, 0.044 MPa, 0.028 MPa, and 0.019 MPa were 51 °C, 50.1 °C, 48.1 °C, and 46.4 °C, respectively. This is because as the pressure inside the vapor chamber decreases, the boiling point of the working fluid also decreases, making it easier for the liquid to evaporate. Therefore, at lower pressures, the internal working fluid can participate in the phase-change heat transfer process in a shorter time, allowing the heat generated by the heat source to be promptly transferred from the evaporator side of the vapor chamber to the condenser side and then to the surrounding air through natural convection.

**Figure 7:**
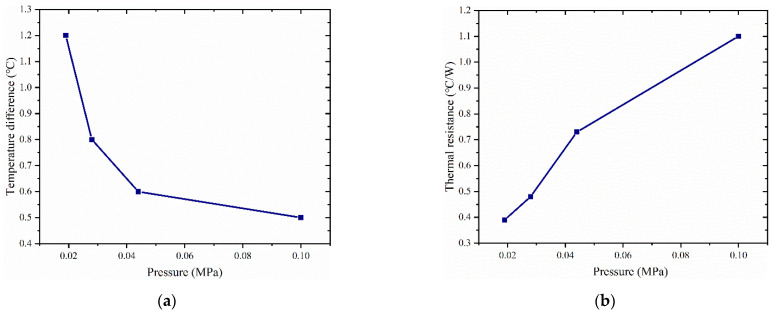
The temperature difference of the surface of the vapor chamber at different pressures: (**a**) maximum temperature difference of the surface of the vapor chamber; (**b**) thermal resistance of the vapor chamber.

Figure 7 shows a vapor chamber’s thermal resistance and maximum surface temperature difference under a thermal load of 5.8 W. As seen from the graph, with decreasing pressure, the surface temperature difference of the vapor chamber increases, and the thermal resistance gradually decreases. For example, at atmospheric pressure, the surface temperature difference and thermal resistance of the vapor chamber are 0.5 °C and 1.1 °C/W, respectively. At a pressure of 0.019 MPa, the maximum surface temperature difference of the vapor chamber is 1.2 °C, and the thermal resistance is 0.39 °C/W. This may be due to the decrease in latent heat of the working fluid and the corresponding decline in boiling point as the pressure decreases. Under the same thermal load, the boiling of the working liquid becomes more intense, which enhances the phase-change heat transfer process in the vapor chamber and reduces thermal resistance.

### 4.3. Impact of Filling Level on Vapor Chamber Performance

**Figure 8:**
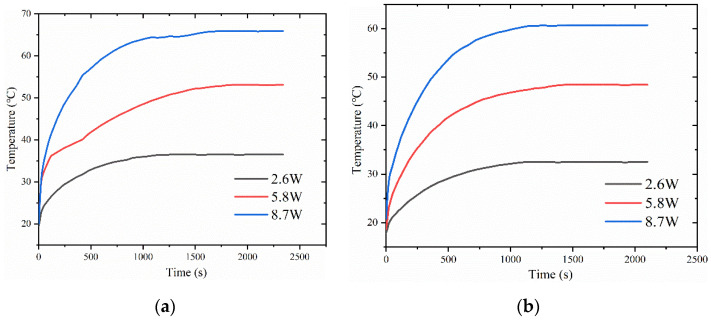

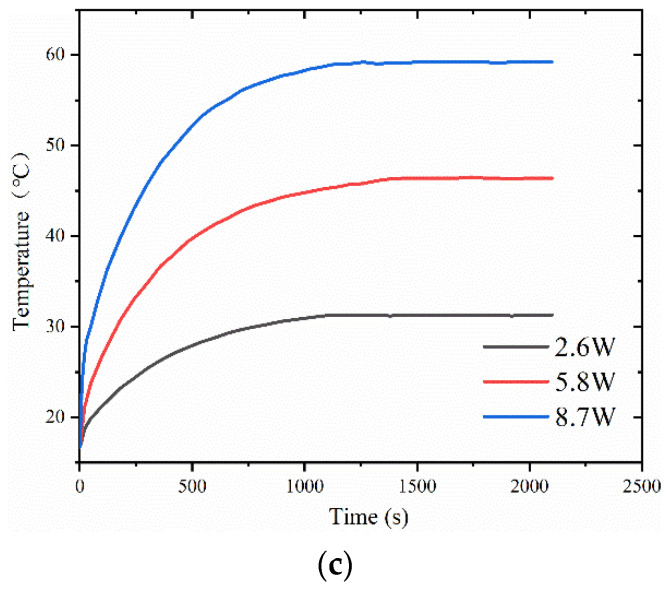
Variation of heat source temperature with time for different liquid filling amounts: (**a**) 0.5 g; (**b**) 1 g; (**c**) 1.5 g.

Figure 8 shows the variation of heat source temperature with time for different liquid filling amounts in the vapor chamber at a pressure of 0.028 MPa inside the vapor chamber. The results show that the temperature of the heat source gradually increases with increasing heat load. However, for a constant heat load, the temperature of the heat source decreases with increasing filling levels. For example, at a heat load of 2.6 W, the temperatures of the heat source for filling levels of 0.5 g, 1 g, and 1.5 g are 36.5 °C, 32.5 °C, and 31.3 °C, respectively. Similarly, at a heat load of 5.8 W, the temperatures of the heat source for filling levels of 0.5 g, 1 g, and 1.5 g are 53.1 °C, 48.4 °C, and 46.4 °C, respectively. At a heat load of 8.7 W, the temperatures of the heat source for filling levels of 0.5 g, 1 g, and 1.5 g are 65.9 °C, 60.7 °C, and 59.2 °C, respectively.

However, we observed a significant temperature change in the heat source when the working fluid filling increased from 0.5 g to 1 g. In contrast, when the filling amount increased from 1 g to 1.5 g, the temperature change of the heat source was not significant, especially at higher heat loads. This may be because a low filling amount resulted in the working fluid being unable to return to the evaporation end during the operation of the vapor chamber, leading to partial drying at the heat source and a decrease in heat transfer efficiency. With an increase in filling amount, enough working fluid was participating in the phase-change heat transfer process, thereby improving heat transfer efficiency.

**Figure 9:**
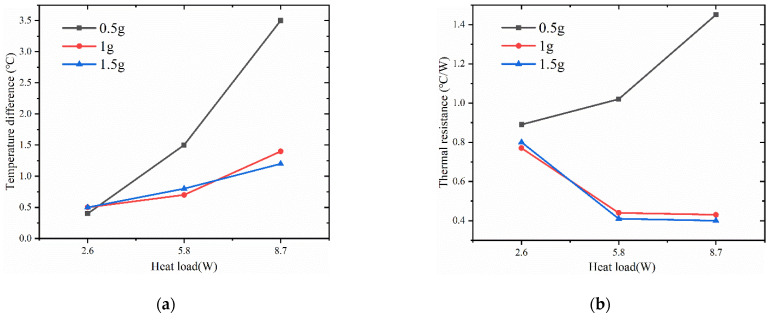
(**a**) Maximum temperature difference of the surface of the condensing end of the vapor chamber under different liquid filling amounts; (**b**) thermal resistance of the vapor chamber under different liquid filling amounts.

In Figure 9a, the thermal resistance of the vapor chamber shows a different trend with increasing thermal load, depending on the filling amount. In the tested range, the thermal resistance increases with increasing thermal load for vapor chamber with 0.5 g filling amount, while it decreases with thermal load for 1 g and 1.5 g filling amount. The reason for this phenomenon may be that when the filling amount is 0.5 g, the increase in heat load causes partial drying inside the vapor chamber, which results in its inability to function correctly and a rapid rise in thermal resistance. At a heat load of 2.6 W, the thermal resistance of the vapor chamber with a filling amount of 0.5 g was significantly higher than that of the other two samples, indicating that the vapor chamber with a filling amount of 0.5 g had already experienced partial drying at this heat load, leading to poor performance. At high heat loads, the thermal resistance of the vapor chamber with filling amounts of 1 g and 1.5 g decreases with increasing heating power because the working fluid inside the vapor chamber can ensure sufficient liquid evaporation and reflux without drying out. In addition, with increasing heating loads, the boiling phenomenon inside the working fluid intensifies, enhancing its heat transfer performance. Meanwhile, we found that at 2.6 W, the vapor chamber with a 1.5 g filling amount has a higher thermal resistance compared to the vapor chamber with a 1 g filling amount. However, at heat loads of 5.8 W and 8.7 W, the vapor chamber with a 1.5 g filling amount exhibits a smaller thermal resistance than the vapor chamber with a 1 g filling amount. This may be because at low heat loads, the working fluid only partially boils, and the higher filling amount of the vapor chamber results in more working liquid in the wick, increasing the flow resistance of the working fluid and making circulation difficult, thereby reducing the heat transfer efficiency and increasing the thermal resistance. However, when working in a high-power environment, the higher heat flux promotes more working fluid to participate in the phase-change cycle, thereby reducing thermal resistance.

From Figure 9b, it can be seen that the maximum temperature difference on the condensation surface of the vapor chamber increases with increasing heat load. The surface temperature difference of the vapor chamber with a filling amount of 0.5 g increases significantly compared to that of the vapor chambers with filling amounts of 1 g and 1.5 g with increasing heating power. This may be because the filling amount is too small, and the internal working fluid is insufficient to consume the heat generated by the high heat flux, resulting in vapor chamber failure.

### 4.4. Antigravity Performance

**Figure 10:**
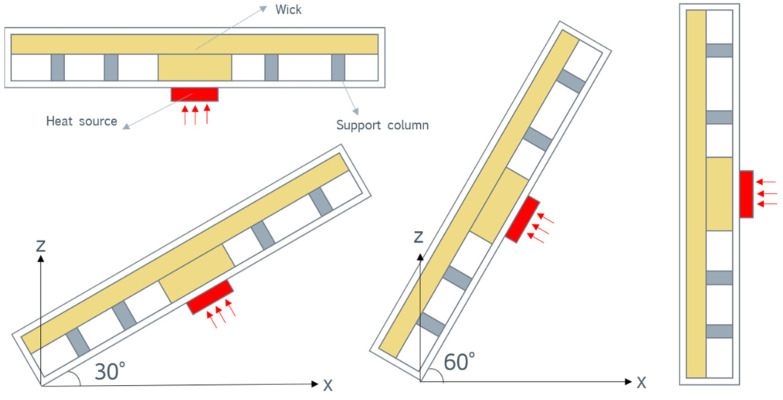
Schematic diagram of the tilt angle of the vapor chamber.

To investigate the antigravity performance of the vapor chamber designed in this paper, the heat transfer performance of the vapor chamber was studied at four different angles. As shown in Figure 10, experiments were conducted on the vapor chamber at four positions: horizontal, inclined at 30°, inclined at 60°, and vertical.

**Figure 11:**
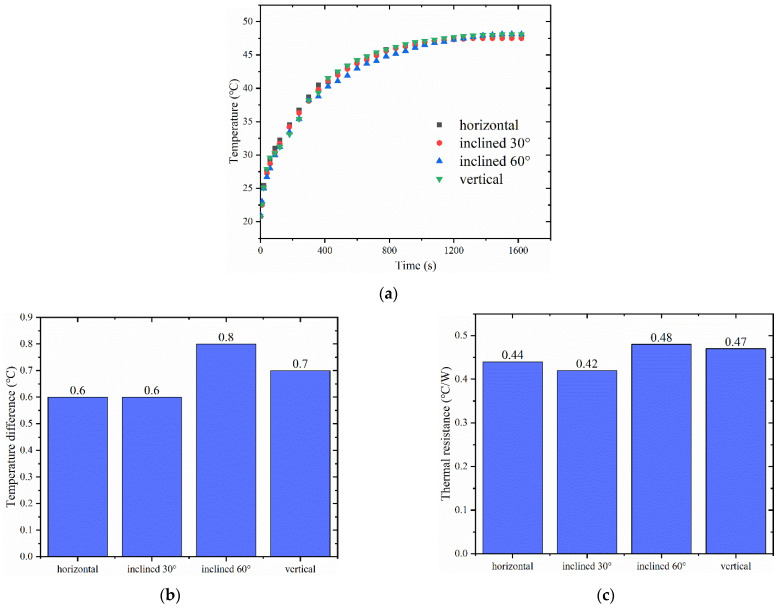
(**a**) Surface temperature of heat source at different tilt angles; (**b**) maximum temperature difference on the surface of vapor chamber at different tilt angles; (**c**) thermal resistance of vapor chamber at different tilt angles.

Figure 11a shows the variation of heat source temperature with time at different tilt angles for a vapor chamber with a working fluid filling of 1 g, internal cavity pressure of 0.028 MPa, and a heat generation rate of 5.8 W. It can be observed that the heat source temperature does not change significantly with the change in tilt angle. Figure 11b,c indicate that the vapor chamber’s surface temperature difference and thermal resistance are not significantly affected by the tilt angle, which shows that the performance has not changed considerably. This is because the vapor chamber’s wick has a full capillary force that allows the working fluid to flow back to the evaporation end, even in the vertical position. Additionally, the tilt angle does not affect the internal vapor flow, which is dominated by molecular forces and can penetrate the entire vapor chamber even in the vertical position. The vapor chamber’s strong heat transfer capability relies on the internal vapor flow, proving that the designed vapor chamber in this study has good antigravity performance.

## 5. Conclusions

In this study, a new vapor chamber was fabricated with an aluminum alloy shell and a cotton wick arranged in a fractal vein-like structure within the vapor chamber. This novel design provides an effective thermal management strategy for some portable electronic devices and offers a new approach to selecting wick materials in vapor chambers. The experimental results demonstrate that the vein-like structure of the wick has good flow and heat transfer performance and exhibits excellent antigravity performance in both horizontal and vertical positions. The study also explores the effects of cavity pressure and working fluid filling on the performance of the vapor chamber. The main conclusions from this study are as follows:

(1) The designed vapor chamber with woven cotton as the wick structure exhibited excellent fluid flow and thermal convection performance. Compared to the other two vapor chambers, this vapor chamber exhibits better performance, with lower thermal resistance and corresponding heat source temperature.
(2) At the filling amount of 0.5 g, the performance of the vapor chamber is the worst. The performance of the vapor chamber does not differ much between the filling amounts of 1 g and 1.5 g, and within the tested range, the thermal resistance decreases with the increase of heat load.
(3) The thermal resistance of the vapor chamber decreases gradually as the pressure in the cavity decreases, and the temperature of the heat source also decreases accordingly.
(4) The designed vapor chamber demonstrated good antigravity performance, and there was no significant change in its heat dissipation performance at four different tilt angles.

In future work, we will explore more suitable manufacturing methods to produce this type of vapor chamber and conduct in-depth research on the working mechanism of this wick. We will also conduct a more in-depth study of this type of vapor chamber.

## Figures and Tables

**Table 1:** Main production materials.

Materials	Role	Size/Diameter
6061 Aluminum alloy	Upper and lower shell plates	160 × 80 × 1 mm
Aluminum wire	Intermediate frame	2 mm
Ethanol	Working fluid	/
Stainless steel liquid-filled tube	Liquid filling, vacuuming	2 mm
Metal glue	Fixed, sealed	/
Silicone sealant	Sealed	/
